# Integrating STEM in elementary classrooms using model-eliciting activities: responsive professional development for mathematics coaches and teachers

**DOI:** 10.1186/s40594-017-0066-3

**Published:** 2017-06-05

**Authors:** Courtney K. Baker, Terrie M. Galanti

**Affiliations:** George Mason University, 4400 University Drive; MS 1E8, Fairfax, VA 22030 USA

**Keywords:** Mathematics education, Model-eliciting activity, Professional development, STEM integration, Mathematics coaching, Design-based implementation research

## Abstract

**Background:**

This research highlights a school-university collaboration to pilot a professional development framework for integrating STEM in K-6 mathematics classrooms in a mid-Atlantic suburban school division. Because mathematics within STEM integration is often characterized as the calculations or the data representations in science classrooms, technology labs, or outside-of-school programs, developing a *reasonable* and *realistic* conceptualization of STEM integration for mathematics teachers and coaches may be especially challenging. Using design-based implementation research, university facilitators worked with eight mathematics teachers and coaches to construct an accessible vision of STEM integration built upon the design features of model-eliciting activities (MEAs). The research team strategized a flexible and fluid professional development that would (1) situate participants’ breadth of experiences on a STEM curriculum integration continuum; (2) elicit a new vision of STEM integration through open-ended mathematics problems with client-driven, real-life contexts; and (3) focus on making mathematics content explicit.

**Results:**

Qualitative analysis of participant discussions and written reflections from a four-day summer institute indicates that the daily tailoring of the professional development design supported an evolving participant envisioning of STEM integration. Opportunities to engage with MEAs as learners, contrast MEAs with problem-based learning and draw from MEA design features to modify existing curricular tasks allowed participants to think more broadly about mathematics content within STEM integration. Participants communicated a readiness to use MEAs as a vehicle for K-6 STEM integration which maintains an important grounding in the teaching realities of grade-level standards and standardized test preparation. They also acknowledged the need for ongoing support as they considered the challenges of curricular pacing and administrative expectation.

**Conclusions:**

The researchers continued to support the school division during monthly academic-year professional development sessions as the teachers and coaches created and enacted prototype lessons. Their shared investment in building STEM integration capacity with a specific focus on mathematics content can offer a model for STEM integration using MEAs that challenges one-size-fits-all professional development, encourages STEM instructional leadership, and promotes mathematical readiness for STEM citizenship and careers.

## Background

School divisions and policymakers across the United States have “embraced” the slogan of STEM (Bybee 2010), but there is limited evidence of theoretical frameworks and a lack of common language for the design and development of sustainable STEM integration in K-12 education [National Academy of Engineering (NAE) & National Research Council Committee (NRC) 2014]. Meeting the educational goals of STEM literacy requires specific attentiveness to each of the four disciplines (Bybee 2010), yet mathematics may be set up to play only a supporting role in STEM integration (Fitzallen 2015) when it is characterized as the calculations or the data representations in science classrooms, technology labs, or outside-of-school programs. STEM should consist of “concrete, identifiable, innovative content accompanied by specific teaching goals and implementation plans” that support mathematics learning (Shaughnessy 2013, p. 324). Achieving a shift in focus within integrated STEM from “the incidental nature of mathematics in learning activities” to “the instrumental nature of the mathematics” (Fitzallen 2015, p. 342) may be especially challenging because of the traditionally siloed nature of mathematics instruction.

Despite standards reform in mathematics and science over the last quarter century, mathematics in particular continues to be taught in isolation with an emphasis on rote skills and memorization (NAE and NRC 2014). Designers of integrated STEM must seek to build a connectedness between mathematics and other subject areas, yet at the same time attend to “learning goals and learning progressions” within mathematics (NAE and NRC 2014, p. 148) and avoid a dilution of mathematics content (Shaughnessy 2013). Without this explicit connectedness, mathematics teachers may perceive STEM integration as an additional instructional requirement that is placed on top of the existing curriculum (Wang et al. 2011).

Extensive professional development is also required to adapt existing instructional materials and practices and to synthesize the possibilities of real-world contexts and grade-level content mastery expectations within STEM integration (Nadelson et al. 2013; NAE and NRC 2014; Stinson et al. 2009). According to Wilson (2011), STEM professional development is often “short, fragmented, ineffective and not designed to meet the specific needs of individual teachers” (as cited in NRC 2011, p. 21). A “one-size fits all” approach to STEM teacher education (Avery and Reeve 2013) may not be productive for teachers of varying backgrounds and experiences, specifically in core academic subjects such as mathematics. As mathematics teachers seek to build upon the ideas they have learned in STEM workshops and seminars, they need targeted and contextual support to grow and sustain these novel practices in their classrooms.

### Purpose of study

With the publication of the standards for mathematical practice in the Common Core State Standards for Mathematics (CCSSM) [National Governors Association Center for Best Practices and Council of Chief State School Officers 2010], there has been growing research interest in the potential and challenges of modeling with mathematics in K-6 classrooms. Modeling allows students to interpret real-world situations in mathematical formats with problem posing (English et al. 2005) and is thus a logical structure for STEM integration.

The university researchers sought to connect the deep educational research base on mathematical modeling and modeling perspectives (Lesh et al. 2000; Lesh and Lehrer 2003) to the practice of integrating STEM in K-6 mathematics classrooms. Model-eliciting activities [(MEAs), Lesh and Doerr 2003], which incorporate client-driven, real-life contexts and engineering design processes in open-ended problem solving (Maiorca and Stohlmann 2014), offer the potential to act as a vehicle for STEM integration. By supporting teaching through problem solving, MEAs offer an entry point for STEM integration in mathematics classrooms yet maintain an important grounding in the teaching realities of grade-level standards by revealing the conceptual strengths and weaknesses of student thinking (Lesh et al. 2000). However, there is limited research on teacher readiness to design and implement MEAs in their classrooms (English 2016; Fitzallen 2015). Exploration of professional development structures that can help teachers to connect research on modeling to classroom practice will advance understandings of STEM integration with a specific focus on mathematics learning (Shaughnessy 2013). The university researchers collaborated with a mid-Atlantic school division to pilot a professional development framework for K-6 mathematics coaches and teachers with an initial summer institute and follow-on monthly support for school-based coaches.

The following research question guided this study: How did university researchers’ design decisions during a summer professional development institute enable K-6 mathematics coaches and teachers to envision MEAs as a vehicle for STEM integration?

### Need for STEM integration in mathematics classrooms

The NAE/NRC Committee on Integrated STEM Education (2014) synthesized research on learning and achievement in existing STEM integration initiatives and challenged designers of future STEM integrated curricula to address the realities that stakeholders face as they try to bridge the intrigue of authentic learning experiences and the reality of building subject-area proficiencies. The committee acknowledged the struggle to define STEM integration.

In educational practice and in research, the term integrated is used loosely and is typically not carefully distinguished from related terms such as connected, unified, interdisciplinary, multidisciplinary, cross-disciplinary, or transdisciplinary. (p. 23)

Moore (2008) defines STEM as a merging of four disciplines to “deepen student understanding of each integration discipline by contextualizing concepts; broaden student understanding of STEM disciplines through exposure to socially and culturally relevant STEM contexts; and increase interest in STEM disciplines by increasing the pathways for students to enter the STEM fields” (as cited in Wang et al. 2011, p. 2). This interdisciplinary framing of STEM integrated curricula focuses on cross-curricular themes in lieu of subject area proficiencies. Yet, for many mathematics teachers, this deep and broad conceptualization of STEM integration may be so distant from their daily understandings of content, curriculum, and pacing that implementation becomes unrealistic (Wang et al. 2011). When STEM integration is perceived as a project or activity that is ancillary to content-specific instruction, it does not offer a pathway to richer mathematics learning.

Vasquez et al. (2013) described STEM curricula on a continuum of increasing levels of integration from multidisciplinary to interdisciplinary to transdisciplinary. A multidisciplinary approach to STEM integration offers a shared theme which students study in separate instructional disciplines; connectivity among disciplines is limited to the theme itself. Interdisciplinary integration offers an additional layer of curricular cohesiveness as learning goals from multiple disciplines are merged within a specific concept. Vasquez and colleagues (2013) emphasized that interdisciplinary integration does not require a team of teachers from the unique disciplines: a single teacher can draw from learning objectives of different disciplines to support deeper learning about a single concept (p. 69). Finally, transdisciplinary integration offers students the opportunity to respond to a real-world essential question using 21st century skills including inquiry processes, problem-solving, critical thinking, creativity, and innovation (English 2016). This continuum allows elementary educators to situate their conceptualizations of mathematics and STEM in flexible ways.

There is a need for additional research on building understanding of mathematics process and content within STEM integration (NAP/NAE, NAE and NRC 2014; English 2016) and a parallel need to explore how STEM integration can promote mathematics literacy as students learn to make “evidence-based decisions involving ethical, economic, and environmental dimensions” (English 2016, p. 4). Although reform curricula and standards have emphasized problem-solving opportunities in mathematics, many teachers still perceive problem solving as an elite activity, accessible only to students who have mastered essential mathematical skills and formulas (Crespo 2003; Wang et al. 2011). For teachers who view mathematics as a set of skills layered upon a real-world context, problem solving may be erroneously perceived as a correct application of tools instead of an integrated instructional experience (Lesh et al. 2000). These perceptions impede opportunities for conceptualization of mathematics teaching within integrated STEM activities. Furthermore, students in underrepresented STEM populations are often denied access to opportunities to practice 21st century skills as they are instead provided with remediation and extra support to gain computational fluency in preparation for standardized college-preparatory assessments. Iversen and Larson (2006) compared college students’ modeling abilities to their standardized mathematics test performance and concluded that students who perform well on traditional assessments do not necessarily perform well in MEAs. Their findings have important implications for access to meaningful opportunities to learn for each and every student in K-6 integrated STEM contexts.

### Conceptual framework

The researchers in this study hypothesized that mathematics coaches and teachers could advance mathematics instruction and problem solving through targeted collaborative opportunities to envision MEAs as a vehicle for STEM integration. MEAs encourage students to iteratively design, develop, and construct mathematical models to solve a problem posed by a client or organization (Lesh et al. 2000). These structures have the potential to support coaches and teachers as they initially envision the contextual affordances of interdisciplinary and transdisciplinary STEM within the boundaries of daily mathematics instruction. Drawing from Stohlmann’s (2013) definition of STEM integration as “an effort for mathematics teachers to use the engineering design process as the structure for students to learn mathematical content along with science concepts through technology-infused activities,” the researchers strategized a flexible and fluid professional development that would (1) place participants’ varied knowledge and experiences on the STEM curriculum integration continuum (Vasquez et al. 2013); (2) elicit a narrower implementation aligned with teacher knowledge and beliefs using MEAs (Lesh et al. 2000; Maiorca and Stohlmann 2014); and (3) focus on making the mathematics content explicit.

### MEAs as a vehicle for STEM integration

MEAs were originally conceptualized as a device to help mathematics education researchers elicit student modeling and to develop expertise about cognition and problem-solving behavior (Lesh et al. 2003). They have since become a tool that can also be used to help teachers and students develop their own competencies (Hamilton et al. 2008). Teachers facilitate student collaboration and problem solving in MEA enactments as students apply their mathematical understandings, explore possible strategies, assess their thinking, compare solutions, and produce a prototype. MEAs support the goals of teaching and learning mathematics within STEM by integrating concepts found inside and outside of mathematics, by encouraging learning through discovery (Magiera 2013) with a specific focus on accessibility to varied learning styles and experiences (Stohlmann 2013) and an orientation toward in-depth solutions and mathematical literacy (English 2015, 2016; Mousoulides 2007). MEAs offer teachers a contextual and content-focused lens on student mathematical thinking (Chamberlin and Moon 2008; Stohlmann 2013) that yields explicit evidence of student learning that is needed in meaningful STEM integration.

Lesh and colleagues (2000) specified six design principles for MEAs, and Magiera (2013) modified the naming and descriptions of these principles in a National Council of Teachers of Mathematics (NCTM) journal article for middle school mathematics teachers which communicated the student-centered intentionality of MEAs.

1. *The model construction principle*: Students must provide an explicit description, explanation, or prediction for a mathematically significant situation.
2. *The reality principle*: Problem solvers must behave like scientists or engineers who are working for a particular client or organization.
3. *The self-assessment principle*: A group of students use criteria embedded in the activity to self-evaluate their work. In turn, they go beyond their initial ways of thinking to create a model that is more robust and more closely aligned with the needs of the client.
4. *The model documentation principle*: Students must produce documentation of their thinking that reveals their mathematical and nonmathematical interpretation of the problem situation.
5. *The generalizability principle*: Students must produce models that are generally useful and could be easily modified and applied to situations that are similar to the one being studied.
6. *The effective prototype principle*: Students use the concepts that underlie the activity to create simple but powerful models for complex situations.

Maiorca and Stohlmann (2014) tailored the original six MEA design principles (Lesh & Doerr, 2003) with a specific orientation toward STEM integration in their definition of four MEA design features: open-ended, client-driven, uses of the engineering design process and mathematics is similar to real life.

Design of STEM integration opportunities can offer open-ended, experiential instruction generally categorized as problem-based learning (NAE & NRC 2014). Within the context of mathematics teaching, problem-based learning (PBL) and MEAs share many characteristics including realistic problems, open-ended tasks, higher-order thinking, metacognitive coaching, self-directed learning, self-assessment, group work, and interconnectedness of disciplines (Chamberlain and Moon 2008). However, MEAs may be more accessible to teachers as an initial investment in STEM integration because they only require one to two class periods and they produce mathematical models which offer teachers a contextual and content-focused lens on student thinking (Chamberlin and Moon 2008; Stohlmann 2013). Hamilton et al. (2008) further differentiate between PBL tasks and MEAs by citing the specific real-world emphasis within MEAs of product as more important than process. They suggest an “important and instructive irony” (p. 12) as MEAs were originally intended to offer a lens on the processes of developing problem-solving competencies. In actuality, the need for a deliverable to meet client needs may evoke more powerful problem solving than a strictly open-ended PBL task.

Researchers have explored the opportunities to learn through modeling in K-6 classrooms (Asempapa 2015; Chan 2008; English and Mousoulides, 2011; Lesh and Doerr, 2003), yet there is limited research on the professional development support that elementary teachers need to facilitate these innovations in teaching and learning. Teaching through MEAs can help elementary students to see the relevance of mathematics in their lives and in society, to experience tasks with high cognitive demand, to build critical thinking skills, and to engage in meaningful mathematics discourse (Asempapa 2015). Chan (2008) outlined three challenges that teachers may face in promoting these learning opportunities. Teachers must “relinquish a fair amount of classroom control” (p. 62) as they strive to promote discourse and to elicit and extend student thinking. They must also be able to see how these experiences connect to preparation and readiness for standardized testing. MEAs present challenges because they differ from teachers’ mathematics learning experiences and require teachers to react and replan in response to creative ideas (Blum and Ferri 2009).

As MEAs necessitate both mastery of lower-level procedural skills and higher-order thinking with a design orientation, they offer the explicit evidence of student mathematical thinking and learning that is needed in meaningful K-12 STEM integration. However, there is a commensurate challenge in developing the necessary expertise to implement MEAs in K-6 classrooms.

### Situating STEM professional development in schools

Educator expertise may be the “key factor” in STEM integration (NAP 2014, p. 115). Professional development is required to support teachers who did not learn mathematics in STEM contexts to build a working knowledge of what STEM integration can look like (Stinson et al. 2009). However, designers of STEM professional development programs face multiple challenges in fostering sustainable STEM pedagogy. Nadelson et al. (2013) described a 3-day professional development institute designed to build STEM inquiry-based teaching capacity in elementary schools using commercially available educational materials. Despite incentives such as college credits and classroom instructional materials, many teachers hesitated to commit to register for the institute. Participants engaged in hands-on engineering activities and worked in grade-level teams to design lessons aligned with district curricula and standards. Although measures of participant confidence and efficacy in STEM teaching showed significant increases, teachers expressed both “excitement and doubt” as they reflected on the potential for integrating these materials with their existing curricula (p. 165). They expressed their continuing view of a disconnect between brick-building STEM engineering activities and the realities of classroom instruction. Participants described their struggle to tie “fun ideas” to the curriculum or to find “activities to fit the curriculum” (p. 165). These responses suggest that connecting hands-on engineering activities to established curriculum and standards may not be enough to build a reasonable and realistic vision of STEM integration.

Professional development programs throughout the United States have enhanced STEM teacher content knowledge, but there is “virtually no research” connecting this new knowledge to classroom teaching and student understandings (NAE and NRC 2014). Research has shown that ongoing mentoring and support (p. 124) and teacher collaboration (p. 125) increase the likelihood of successful STEM integration (NAE and NRC, 2014). Additionally, professional development that is both “site-based” and “curriculum-linked” (Penuel et al. 2007, p. 928) is theorized to improve teacher enactment of reform-oriented instruction.

School-based mathematics coaches are positioned to bridge potential gaps between STEM education seminars and conferences which are inherently distant from specific school contexts and day-to-day classroom obligations. As division teams of coaches and teachers engage in professional development that integrates mathematics learning and engineering design principles within their own curricular materials, they can become a sustained STEM leadership presence in their school communities. They can both model and expect longitudinal enactment through job-embedded professional development that is more likely to promote not only STEM teacher knowledge but more importantly STEM teacher practice.

## Methods

This study analyzes the professional development design decisions of the university researchers as they collaborated with mathematics teachers and coaches to construct a reasonable and realistic vision of STEM integration using MEAs. The research team used a design-based implementation research (DBIR) methodology to bridge a growing body of research on MEAs as a tool to help students to become better problem solvers (Hamilton et al. 2008) to the practice of STEM integration within a specific school division. DBIR allowed the team to draw upon the research on specific enactments of MEAs as tools in mathematics and engineering education. Additionally, DBIR supported both the theorization and practical support that teachers and coaches would need to implement these tools (Penuel et al. 2011) to create more meaningful mathematics opportunities to learn in STEM integration contexts.

DBIR is comprised of four principles that align with the goal of constructing a responsive professional development model for integrated STEM in mathematics classrooms: (1) a focus on persistent problems of practice from multiple stakeholders’ perspectives; (2) a concern with developing capacity for sustaining change in systems; (3) a concern with developing theory and knowledge related to both classroom learning and implementation through systematic inquiry; and (4) a commitment to iterative, collaborative design (Fishman et al. 2013; Penuel et al. 2011; Leary et al. 2016).

The university researchers and the division math supervisor (DMS) collaborated to theorize a system of support in response to an administrative initiative to integrate quarterly STEM tasks at each grade level. The DMS had requested support from the university due to past experiences with the university researchers’ professional development in ambitious mathematics instruction. The DMS sought to leverage the university’s mathematics education expertise in conjunction with coaching knowledge to highlight modeling and to bring mathematics to the forefront of STEM integration. The university researchers wanted to support the DMS’s vision but also protect and sustain the established working relationship. The negotiation resulted in a professional development structure in which mathematics coaches from each of the eight division schools would attend a 4-day summer institute on STEM integration and receive ongoing professional development throughout the year in pre-established monthly coaching meetings.

This professional development structure was designed to unify the mathematics coaches by targeting specific leadership needs and incorporating K-12 vertical articulation, prior. Prior to the professional development, the DMS informed the university researchers that only two of the eight mathematics coaches would be able to attend. As a result, the university researchers revised the professional development to meet the needs of a new K-6 audience including not only mathematics coaches but also a special educator, an interventionist, and classroom teachers. This change in membership provided space for additional iterations within the implementation research to gather evidence of how a broader range of school division stakeholders would experience and envision integrated STEM enactments.

The initial professional development was centered on a STEM disciplinary continuum (Vasquez et al. 2013) to allow mathematics coaches to purposefully apply MEA design principles in the creation or adaptation of math tasks to meet the teacher’s individual needs. However, in order to effectively respond to the new audience, it was important to have the design flexibility to respond to a broader range of content and pedagogical expertise. The researchers and participants engaged in a shared intellectual journey to work toward the goals of STEM integration in a manner that was both reasonable and realistic for K-6 educators. The researchers used purposeful groupings and differentiated questions to encourage collaboration among participants and to connect STEM integration to the individual contexts within schools. The broader participation of mathematics educators allowed the development of a more cohesive theorization of MEAs as a vehicle for STEM learning and implementation. Iterative reflection and redesign occurred after each full day of professional development to address stakeholder concerns and to challenge their growing understanding of the roles MEAs could play within STEM integration.

### Participants and setting

The participants were four classroom teachers, three mathematics coaches, one elementary mathematics interventionist, one middle grades special educator, and the DMS. These educators were self-selected to further their understanding of STEM integration in mathematics as part of a 3-week summer mathematics curriculum writing experience. As a result, they were more likely to find STEM integration worthwhile and had the potential to become STEM teacher leaders. These individuals were interested in improving mathematics instruction through the development of curricular materials. Several had previously attended STEM institutes or professional development conferences outside the school division.

With the exception of the high school, each of the eight schools within this suburban school division had participant representation from either a mathematics coach or teacher. Within this school division, 73% of students were traditionally underserved (African American, American Indian, Hispanic/Latino) and 53% of students were economically disadvantaged (State Department of Education, 2015). Each of the five elementary schools in this division received additional Title I government funding to ensure that all children met academic standards.

### Data sources

Multiple qualitative data sources were used to support the iterative design and development of the professional development model. Participants responded to daily writing prompts that allowed researchers to analyze evolving perceptions of STEM integration. These reflections were centered on (1) participants’ perceptions and understandings of STEM; (2) connections of participants’ specific contexts and roles to STEM integration; (3) the affordances and challenges MEAs offered to mathematics teaching and coaching; and (4) development of reasonable and realistic goals for the upcoming school year that involved the integration of MEAs. Additionally, discussions within the professional development were audio recorded to capture the dynamic nature of conversations and preserve the integrity of the participants’ experiences with and perceptions of the institute content. Finally, each participant also completed the Teacher Efficacy and Attitudes Toward STEM Survey for Elementary Teachers (T-STEM) (Friday Institute for Educational Innovation, 2012).

The researchers created task adaptation charts allowing participants to evaluate the strengths of existing mathematics tasks in relation to MEA design features and to define contextual challenges for implementation. Each day the researchers debriefed on the evidence gathered from their collective monitoring while maintaining the multiple iterations of the professional development materials.

### Data analysis

Data gathered was qualitatively analyzed to inform real-time changes in the professional development structure and to support longitudinal evaluations of changing conceptualizations of STEM integration. University researchers examined participant daily writing prompts at the end of each session and modified the professional development structure in response to participant questions and reactions. This process maximized stakeholder involvement and negotiation in design decisions (Fishman et al. 2013; Penuel et al. 2011) and targeted specific participant needs through the authentic integration of theories and research. Examples of specific modifications from daily written reflections and the researcher debriefs included the construction of an MEA framework using the four MEA design features (Maiorca and Stohlmann 2014) in lieu of the more complex MEA design principles (Lesh et al. 2000; Magiera 2013), the research on the distinction between MEAs and PBL tasks (Chamberlain and Moon 2008), and the purposeful tabling of discussions of assessment within MEAs until later in the academic year.

Due to the short nature of the summer institute, the T-STEM survey was not used as a pre- and post-assessment. Instead, participant responses helped to frame their dispositions towards STEM integration. Each of the participants came to the summer institute with high mathematics teaching efficacy. With regard to teacher leadership, 100% of participants either agreed or strongly agreed that it is important for teachers to take responsibility for all students’ learning, communicate vision to students, use a variety of data to evaluate progress, use a variety of data to set goals, safe and orderly environment, and empower students.

Additionally, all participants felt that students should have opportunities to incorporate each of the specified 21st century skills.

Final reflection prompts given at the end of the professional development provided feedback on what specific information the participants found useful and what still challenged their thinking with regard to using MEAs to integrate STEM. First cycle coding of the daily prompts, the session audio recordings, and the final reflection yielded a set of process codes (Saldaña 2016) to connote the actions and reactions of both the participants and the researchers during the professional development. The reduction of these process codes into categories during second cycle coding led to the emergence of six themes for participation elicited by specific professional development design decisions. This analysis of actions and reactions provided evidence of a central or core category (Strauss and Corbin 1998, p. 146) of emergent yet guarded teacher readiness to engage their students in modeling activities.

## Results and discussion

The summer institute provided a forum in which participants cooperatively explored sustainable STEM integration using the design principles of MEAs. To advance the collective thinking of participants with a range of roles and responsibilities in their school division, researchers iteratively adapted the initial professional development design to support broader thinking about the possibilities of MEAs for STEM integration. By the end of the professional development, the participants described a readiness to draw on MEA design features (Maiorca and Stohlmann 2014) in lieu of the more complex design principles (Lesh et al. 2000) in their curriculum design and a recognition of a place for mathematics content and curriculum within STEM. Analysis of evidence from participant reflections and conversations during and after the institute revealed a teacher growth in envisioning MEAs as a vehicle for STEM integration informed by a sequence of professional development design decisions.

### Action #1: experiencing an existing MEA as a model of STEM integration

On day 1 of the summer institute, the university researchers encouraged the teachers to situate their existing broader understandings of STEM on the disciplinary continuum (Vasquez et al. 2013) before introducing MEAs. The definition of MEAs offered by Maiorca and Stohlmann in their 2014 NCTM annual conference presentation titled “The How and Why of STEM Model-Eliciting Activities” provided an accessible set of four design features (open-ended, client-driven, math similar to real-life, and engineering design process) which the participants referenced and cited for the duration of the institute. These practitioner-friendly constructs were a crucial bridge from the researcher language of MEA design principles to participant envisioning.

**Fig. 1:**
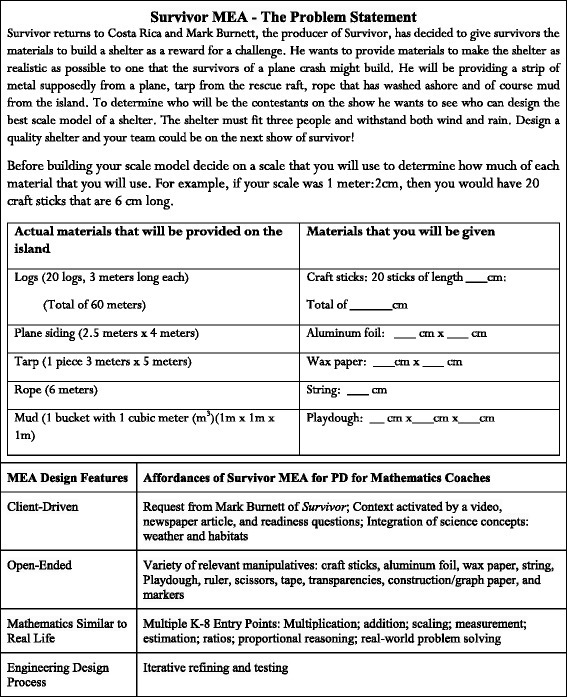
Survivor MEA summary and design feature alignment (adapted from http://wordpress.unlvcoe.net/wordpress/wp-content/uploads/2013/01/Survivor-MEA-Teacher-Materials.pdf)

Maiorca and Stohlmann included the “Survivor MEA” (Fig. 1) from their University of Nevada Las Vegas online library in their NCTM presentation. The university researchers anticipated that this MEA, with its accompanying publically available instructional resources, would offer an engaging, relatable context for the participants as they experienced their first mathematics-focused STEM integration task.

**Fig. 2:**
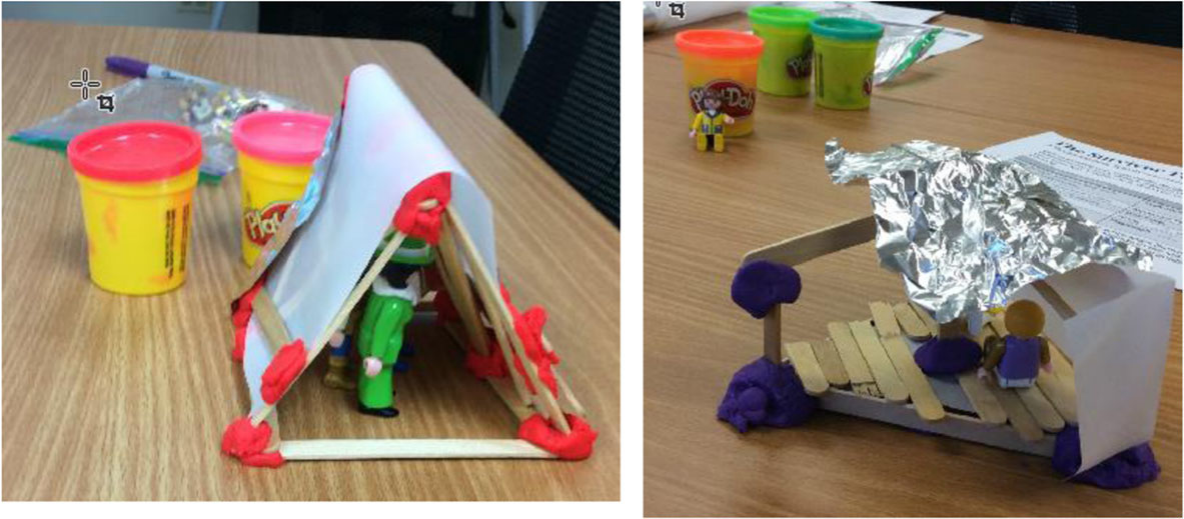
Survivor MEA—physical models of water-resistant shelters

Participants were immediately engaged in the proportional reasoning needed to determine the amount of materials they would be able to use for their prototype. Participant approaches to creating a workable scale and using the correct amount of construction materials varied from precise metric measurements and mathematical calculations to estimation with craft sticks as a unit of measure. After participants had measured materials in hand, they engaged in scientific reasoning and iterative engineering design to create and recreate shelters that met client constraints (Fig. 2). As the teams shared their mathematical and physical models, they discussed what this task could look like at various grade levels within their classrooms and schools.

### Action #2: questioning a place for MEAs in STEM integration

At the end of day 1, Helen, a 2nd grade classroom teacher, expressed skepticism that MEAs were simply another form of PBL that she had learned about in a prior professional development session. In her opening reflection on “What is STEM?,” she recalled her experiences as an engineering camp teacher and described STEM as a “way to create students who will grow to be 21st century thinkers…people who will have a better shot in our ever-evolving, innovative world.” The day 2 presentation was modified to draw from research on similarities and differences of PBL and MEAs (Chamberlin and Moon 2008).

MEAs may be more challenging in mathematics than PBL tasks and they link to mathematical content areas often better than PBL tasks do. Hence, it may be easier for instructors to identify what mathematics is learned by students with MEAs with greater precision than with PBLs. (pp. 19-20)

The researchers emphasized the specific affordance of MEAs as mathematical models providing evidence of student thinking centered on targeted mathematics content. The participants confirmed that this purposeful introduction of research literature helped to clarify the distinction between MEAs and other forms of open-ended STEM teaching.

### Action #3: analyzing a rich mathematics task using MEA features

**Fig. 3:**
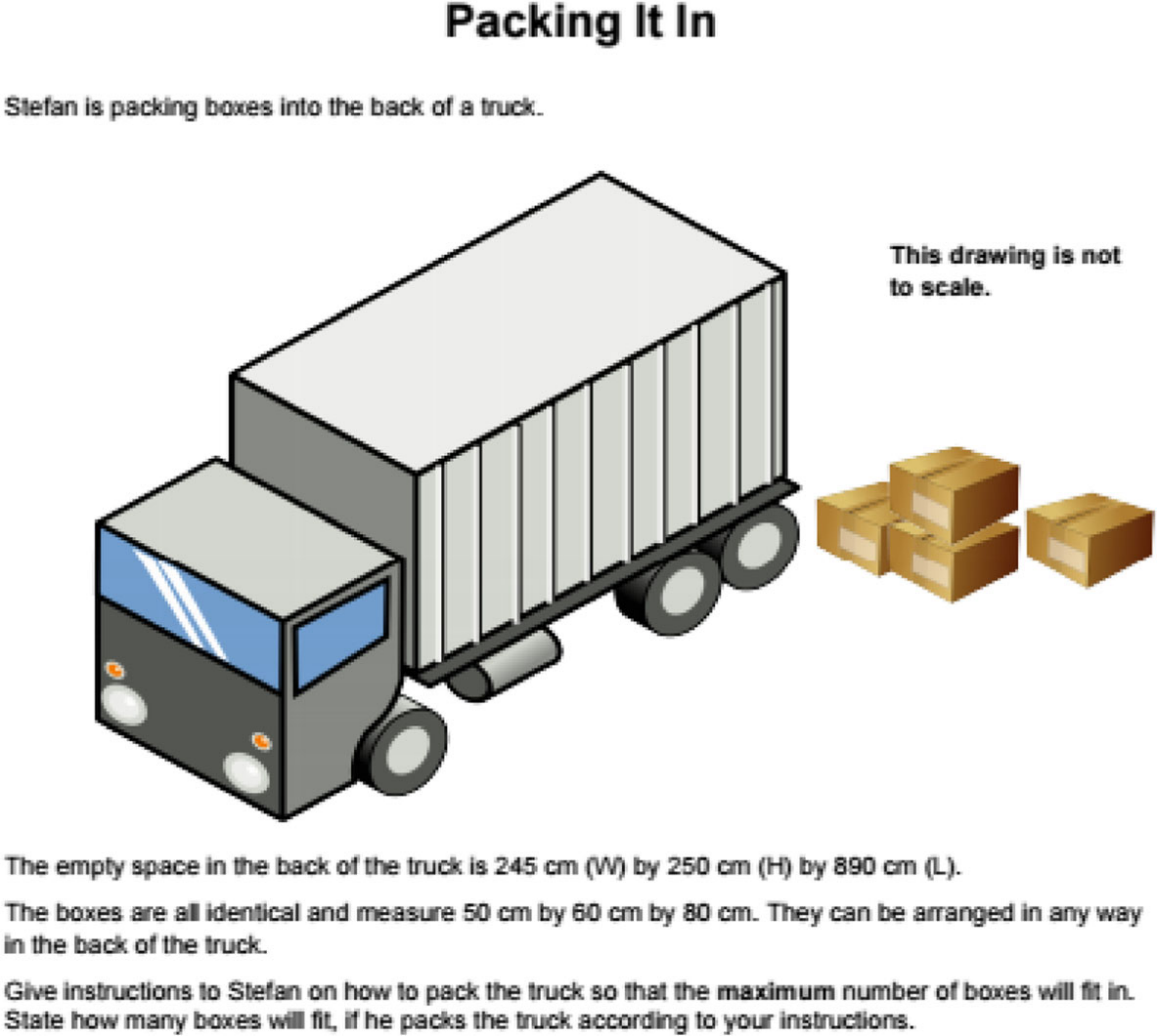
​Adapted from Mathematics Assessment Resource Service. (2016). Using space efficiently: packing a truck. retrieved from http://map.mathshell.org/lessons.php?collection=8

In the next phase of the journey toward conceptualization MEAs as a framework for STEM integration in mathematics classrooms, groups analyzed the Mathematics Assessment Project 6th grade task titled “Using Space Efficiently: Packing a Truck” (Fig. 3). They devised solutions in order to evaluate the strengths of the task and to propose adaptations in relation to the MEA features. The researchers selected this task because it was not only mathematically accessible to students and teachers over a broad range of grade levels but also because it was representative of widely available online resources for rich mathematics problems with real-world contexts.

While the original problem elicited a single correct answer for the most efficient way to pack the truck with boxes of specific dimensions, the researchers modified the question to elicit a model instead of a single solution for packing the truck. Participants proposed contextual questions for Stefan about the content of the boxes and the stackability of the boxes to meaningfully initiate the engineering design process. Coaches and teachers collaborated to analyze this more open problem by exploring criteria and constraints for designing a solution.

The researchers also provided a task adaptation template to support the participants in reflecting on ways to further open this mathematics task and to incorporate engineering design as students proposed an arrangement of boxes for Stefan’s truck. A team of two upper-elementary teachers and one middle school mathematics coach described strengths and adaptations as they imagined a place for the “Packing It In” task in their classrooms (Table 1).

**Table 1:** Completed task adaptation chart for the “Packing It In” task

MEA features	Task strengths	Adaptations required
Open ended	Ambiguity of the expected solution is a strength Giving advice to a client	Allowing students to choose own dimensions. Writing dimensions on truck = visual help
Client driven	Giving advice to a client Helping Stefan pack	Background on Stefan What is he packing? Pack: back → front/bottom → top Stack boxes on top of each other
Math similar to real life	Giving advice to a client Actual dimensions of truck and boxes	Providing the dimensions of the truck bed and asking what dimension boxes would fit most ideally in a truck of that volume (realistic) Manipulative: snap cubes, base 10, Snap cubes are more ideal, can snap more together, and make a scale Graph paper—make bed of truck
Engineering design process	Giving advice to a client	Create the box—using manipulatives Create change as they go along Making more focused in design process.

### Action #4: adapting existing curriculum using MEA features

In order to allow the coaches and teachers to build the critical connection between existing mathematics curriculum and the potential for more open-ended problem solving and modeling, the research team purposefully selected teams of coaches and teachers to collaborate to modify existing division-level common unit assignments. Coaches and teachers were encouraged to draw upon their experiences with the Survivor MEA and the “Packing It In” task to incorporate any or all of the four features of MEAs (Maiorca and Stohlmann 2014) with a focus on realistic goal setting for themselves, for their teaching teams, and for their schools.

#### 7th grade task: “Creating a Mosaic”

**Fig. 4:**
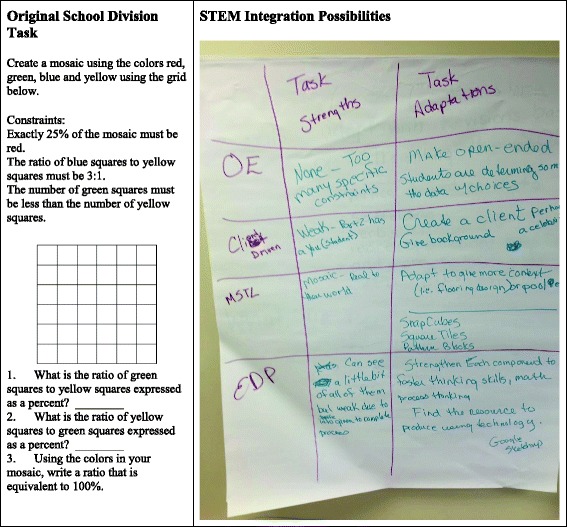
Small group modifications for the 7th grade mosaic design task

One of the teams selected an existing 7th grade task on mosaic design (Fig. 4). They imagined a celebrity client, student specification and representation of tile color relationships, use of manipulatives (tools) to support the engineering of a prototype design, and creation of a Google Sketchup (technology) product.

#### 3rd grade task: “A Day at the Zoo”

**Fig. 5:**
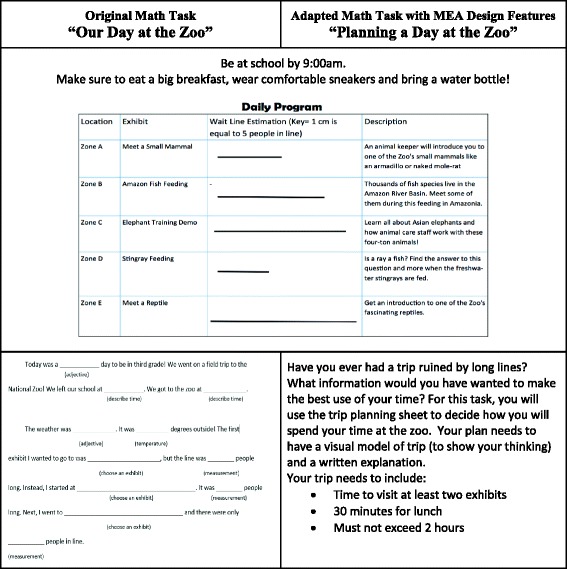
Adaptations for 3rd grade “A Day at the Zoo” task

A second team consisting of Clara, a 3rd grade classroom teacher, and Hannah, a K-6 mathematics coach, adapted an existing task from a grade-level unit assessment. In the original task “A Day at the Zoo” (Fig. 5), students measured line segments and used a pre-identified key to calculate how long they would spend in line for specified zoo attractions. Clara and Hannah used the task adaptation chart to describe the strengths of the original task with respect to the MEA design features. The context provided mathematics *similar to real life*. The original task also included a modified written response in which students could complete a cloze passage centered on the students’ “field trip” by filling in blank spaces with the chosen order of the five attractions.

Using the task adaptation chart, Clara and Hannah identified potential adaptations to further align the task with MEA design features. They saw that the ordering of the five attractions was the extent of the open-endedness and that the mathematical rigor was limited to the calculation of elapsed time for the sequence of attractions. They increased the *open-endedness* of the task by asking the students to engage in more rigorous mathematical reasoning. In their modifications, students would experience the *engineering design process* by creating models and testing different combinations of attractions meeting their new time constraints. The adaptations maintained the mathematics at the forefront of the task through a more creative use of elapsed time calculations. Although the resulting task may not have satisfied all research-based design principles of MEAs, the task adaptation chart with the four MEA features offered an accessible framework to purposefully enhance a rich mathematical task and to work toward a reasonable teacher envisioning of STEM integration.

Clara was eager to implement the adapted task within the first weeks of school to see how MEA modifications would integrate STEM in their 3rd-grade mathematics curriculum. Hannah, a mathematics coach located at a different school, joined Clara to support implementation of this task. Clara’s e-mail correspondence offered a practitioner perspective on the affordances of producing a model within a rich mathematics task.

Due to the way that it was set up, I believe the task gave me more insight into what the students’ thinking was. Because students had to show all of their thinking and work, as well as include a letter that takes you through their process, I can better identify where they are at.

Clara further reflected on how she could incorporate her next adapted task to better fit her students’ needs. She advanced her envisioning of STEM integration with more equitable access to problem solving and expressed an enjoyment of these new opportunities to learn.

I don’t think their MEAs were a true representation because it had to be so teacher directed. I think moving forward I will use more picture cues in the instructions and throughout the assignment…I would love to see [my English Language Learners] really show what they know and it’s not fair to them if they can’t because of a language barrier. I think this is where I will be making the most adjustment moving forward … For a self-proclaimed ‘not math’ person, I am enjoying this math.

### Action #5: envisioning the affordances of implementing MEAs

At the beginning of the professional development, participants reflected on the prompt, “What is STEM? What does it mean to you?” Their responses suggested grand visions of the possibilities of STEM that were consistent with the multi- or transdisciplinary integration emphasizing long-term projects and 21st century skills. One of the coaches expressed her feeling of distance from a reasonable STEM implementation and her desire for a more grounded vision.

I don’t even try to define this by letter given the variety of perspectives in my school as well as in the division and in the larger education community in general. What I hope for is that integrated STEM brings about what I believe teaching and learning should look like. (Elizabeth, 6-8 Mathematics Coach)

The researchers’ professional development focus on practitioner-friendly exploration of MEAs provided an important juxtaposition of the real daily demands of mathematics curricula and a seemingly less realistic unbounded creative project. The teachers expressed surprise that a “product” of an integrated STEM task built upon MEA design principles was not narrowly defined as a single solution and that varying representations of iterative student thinking should be equally valued. By evaluating the strengths and challenges of publicly available MEA resources and then modifying division assignments, teachers and coaches expressed an excitement and readiness to take risks with modeling experiences. Katharine, a 5th-grade special education teacher, perceived that STEM was “not something else to implement” and that she could focus on “quality over quantity.” Other participants elaborated on this recognition that MEAs could be a vehicle for content instruction.

The first week of school, I am going to do an MEA. What a neat way to see how my 6th graders will approach a problem. I’m planning on giving them the same supplies and seeing how they attack it…And taking myself out of it. Not introducing any of the vocabulary and the components. Just seeing what they already know. Then I’ll know where they are and where I need to go. That’s going to help me with the pacing and meeting the needs of my students as individuals. (MacKenzie, 6th Grade Mathematics Teacher)

Allowing [students] to think, reason and solve this type of problem at the beginning of a concept makes them feel more successful to make these connections later. This is going to be a shift in thinking for us – to incorporate these MEAs within the unit and not save them for the end as an assessment. (Hannah, K-6 Mathematics Coach)

### Action #6: reflecting on the challenges of implementing MEAs

Despite their enthusiasm about the instructional potential of MEAs, the mathematics coaches and teachers also acknowledged obstacles to broad implementation with respect to pacing and administrator expectations. They expressed concern about MEAs in terms of available time and teacher buy-in.

I think about how many times I’ve encountered teachers who think ‘How can I get the math in?’ How are we going to get teachers on board with this, if there’s this pacing guide that emphasizes completion of a math concept in three days? (Mackenzie, 6th Grade Math Teacher)

I hesitate about administrators knowing [about MEAs], because I worry about them expecting more from us. It will become an initiative and I don’t want to have to send a report every month of my implementation. I don’t want to do that. (Cora; 3rd Grade Teacher)

On the final day of the workshop, teachers revisited the initial reflection question about their individual understandings of STEM. Participants maintained their valuation of open-ended, student-centered creative thinking and collaboration but recognized that the construction of a mathematical model in response to client needs could evoke solutions that aligned with their curricula.

Seeing those four [MEA] design features over and over again took a lot of the pressure away because we were allowed to just focus on those four things. And every single day you can implement aspects of those design features into your math instruction. Too often you come to [professional development] like this and make a beautiful overly-ambitious thing that brings with it stress and a sense of impossibility. This PD purposefully put us with our grade-level teams, and we took something that we have to do already and worked to improve it in a way that made sense to us. (Cora, 3rd Grade Teacher)

## Conclusions

The purposeful design of this professional development supported coaches and teachers with varying levels of experience and content expertise to make explicit connections between the constraints of curriculum and the possibilities for open-ended problem solving within real-world contexts. The construction of a *reasonable* and *realistic* STEM orientation for teachers is critical as the education community looks toward broader engagement with STEM in classrooms. During the first 2 days of the summer institute, participants were challenged to experience an MEA and to question their conceptualizations of STEM from prior professional development experiences. University facilitators engaged in real-time iterations of planning, reflecting, and revising as they gauged participant perceptions and responded to participant challenges to connect research to practice. When teachers analyzed and adapted rich mathematics tasks to incorporate MEA design features, they envisioned multiple entry points within these problem-solving structures and reflected on implementation challenges.

The summer institute described in this study was designed to initially explore how teachers envisioned MEAs as a vehicle for STEM integration. Limitations of this study include a small sample size, one representative from each K-6 school setting, self-selected individuals, who entered the professional development experiences already believing in teacher leadership and 21st century skills, and participants who possessed high efficacy with regards to mathematics teaching and learning. The participants were individuals who continually sought out opportunities to learn and reflect on their practice. However, despite the confidence and leadership efficacy each participant possessed, several barriers arose that impacted their envisionment of MEAs as STEM integration in classroom settings.

School contexts, administrator expectations, and assessment-driven cultures must inform the ongoing negotiation of STEM implementation. The four MEA design features were flexible enough to allow the coaches and teachers within this study to adapt unit assignments in a variety of ways. Although this study critically examined only a small population of mathematics teachers and coaches during one summer institute, the lessons learned from these experiences can inform future research in STEM professional development design to implement MEAs in mathematics classroom instruction. Additional research on iteration and revision of classroom MEAs through Lesson Study [(LS), Lewis et al. 2006)] would extend this model of situated, context-driven, coach facilitated professional development.

The challenges that the teachers predicted in enacting MEAs evoked an expressed need for ongoing school-based support and reflection on enactments.

We need someone to almost oversee us, someone to help us through the process to bounce ideas off of, a collaborative peer or buddy in the building to work through this and provide feedback, ‘How did it go? What did you try this year? How was everything changed or modified? How did that process go?’ Really focusing on the process, not the product. That’s the important part (Clara, 3rd grade teacher)

University researchers are currently facilitating monthly academic-year professional development for mathematics coaches from all K-6 schools within the division to pilot MEAs in classrooms and to analyze evidence of opportunities for mathematical thinking. This continuing investment will build upon the summer institute model for STEM integration and respond to the expressed needs of the participants. The sequential actions of experiencing, questioning, analyzing, adapting, envisioning, and reflecting on MEAs as a vehicle for STEM integration in mathematics classrooms will inform additional iterations of professional development that is tailored to the context and readiness of its recipients.

## Abbreviations

DBIR: Design-based implementation research
MEA: Model-eliciting activities
NAE: National Academy of Engineering
NCTM: National Council of Teachers of Mathematics
NRC: National Research Council
PBL: Problem-based learning
PD: Professional development
STEM: Science, Engineering, Technology and Mathematics
